# All-Around Electromagnetic Wave Absorber Based on Ni–Zn Ferrite

**DOI:** 10.1021/acsami.4c06498

**Published:** 2024-06-20

**Authors:** Dipika Mandal, Bishal Bhandari, Suraj V. Mullurkara, Paul R. Ohodnicki

**Affiliations:** Department of Mechanical Engineering and Materials Science, University of Pittsburgh, Pittsburgh, Pennsylvania 15260, United States

**Keywords:** microwave absorber, EM wave absorption, ferrite, impedance matching, reflection loss

## Abstract

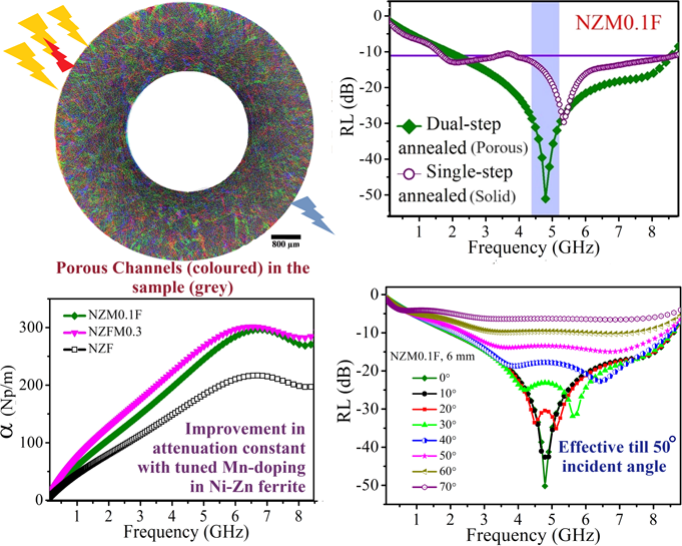

Exploring a convenient, scalable, yet effective broadband electromagnetic wave absorber (EMA) in the gigahertz (GHz) region is of high interest today to quench its expanding demand. Ni–Zn ferrite is considered as a potential EMA; however, their performance study as a scalable effective millimeter-length absorber is still limited. Herein, we investigated EM wave attenuation properties of Ni_0.5_Zn_0.5_Fe_2_O_4_ (NZF) samples substituting Mn ion in place of Fe^3+^ as well as Zn^2+^ within a widely used frequency range of 0.1–9 GHz. Through composition optimization, Ni_0.5_Zn_0.4_Mn_0.1_Fe_2_O_4_ (NZM0.1F) EMA demonstrates excellent microwave absorption performance accompanied by simultaneous maximum reflection loss (RL) of −50.2 dB and wide BW of 6.8 GHz (with RL < −10 dB, i.e., attenuation >90%) at an optimum thickness of 6 mm. Moreover, the attenuation constant significantly increases from ∼217 to 301 Np/m with Mn doping. The key contribution arises from magnetic–dielectric properties synergy along with enhanced dielectric and magnetic losses owing to cation chemistry and site occupation in spinel NZF. In addition, porosity is induced in the system by a controlled two-step heat treatment process that promotes total loss with multiple internal reflections of the EM wave. Furthermore, RL is simulated by varying incident EM wave angles for the NZM0.1F sample displaying its angle insensitivity up to 50°. Our results reveal NZM0.1F as a futuristic environment-friendly microwave absorber material that is suitable for practical high-frequency applications.

## I Introduction

Electromagnetic interference (EMI) arises as an inevitable offshoot from the newly emerged fifth-generation (5G) devices as well as extensively used Radar and satellite communications.^1,2^ However, EMI or electromagnetic (EM) wave pollution not only adversely impacts electronic operations creating unwanted noises but is also considered as a potential environmental hazard.^3,4^ Therefore, demand for an all-around efficient electromagnetic absorber (EMA) is ever-expanding to cut down the EM wave pollution.^5,6^ Moreover, EMA can also shield EM waves to prevent information theft from becoming essential in defense and military fields.^7−9^ In this context, ferrites with their synergistic magnetic and electrical properties are well known to be highly promising EMA components compared with other unary electrical or magnetic materials.^10−12^ Proper tuning of ferrite properties such as permeability (μ) and permittivity (ε) can lead to broader bandwidth (BW) and induce better impedance matching (i.e., |*Z*_in_/*Z*_0_| ∼ 1), thus ensuring higher reflection loss (RL).^1,3,13^ Further, a high value of ε and μ is desirable to enhance the wave attenuation and lessen the thickness (*t*) of the absorbing material.^14^ To achieve tuning, chemical doping, particle size, and morphology control, layering of EMAs are some of the popular techniques.^15−18^ For instance, Gorai et al. observed an optimum RL of −47.0 dB for a bilayered CoFe_2_O_4_-coated MnFe_2_O_4_ nanohollow spheres increasing μ of the material closer to its ε and thus improving impedance matching in ferrites.^19^ Again, a thickness-dependent electromagnetic properties study on Mn–Zn ferrite has been performed by Yang et al., resulting the maximum RL of −22 dB to its corresponding matching *t* ∼ 1.5 mm.^20^

Despite growing searches for a low-cost, lightweight, and effective broadband EMA, there is a significant deficiency for mm-sized absorbers corresponding to their huge demand, and scalability or mass production of EMAs is majorly considered as a concern.^21^ Another limitation in this research area is incident EM wave angle insensitivity studies on ferrite-based absorbing materials.^21,22^ Herein, we have mainly targeted these technical gaps by synthesizing Mn-doped Ni–Zn ferrite in a modified facile solid-state route and investigating their EM wave attenuation properties along with sample thickness and incident wave angle dependence studies. In this aspect, spinel ferrimagnet Ni–Zn ferrite (NZF) is recognized to be suitable as microwave ferrites as a high μ value sustains up to GHz range in NZF from the viewpoint of Snoek’s limitation rule for permeability dispersion.^23−25^ Interestingly, among a few previous studies on NZF EMAs, Derakhshani et al. reported the minimum RL value of −36.1 dB with the effective absorption BW of 6.7 GHz for single-layer bulk NZF.^7^ Outcome of this study suggests that further balance in μ and ε values is required to prevail impedance matching for a wider range. Selective doping of strong magnetic Mn in NZF is a proven strategy to enhance its magnetic permeability and substantially influence dielectric polarization by cation redistribution in the structure.^26^ This cationic rearrangement will change dipole configuration as well as exchange interaction between the tetrahedral (A) and octahedral (B) sites, which in turn tunes ε and μ values of the material.^20^ Furthermore, the total loss can be augmented by inducing mm-length porous channels and additional interfaces to the bulk ferrite, which elongates the path length of the microwave through internal reflection and enhances total loss in the material.^27−29^

**Figure 1:**
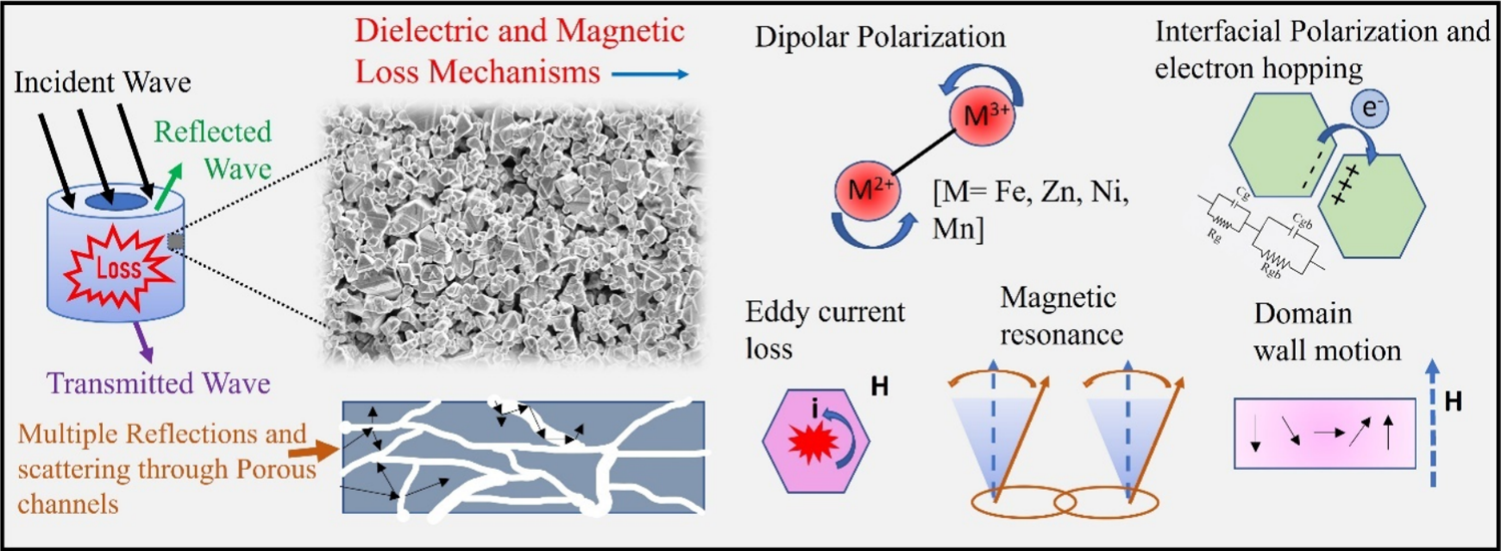
Schematic representation showing the responsible factors for excellent EM wave absorption in Mn-doped NZF cores.

In this study, two sets of Mn-ion-substituted μm-sized Ni_0.5_Zn_0.5_Fe_2_O_4_ samples (I. Ni_0.5_Zn_0.5_Fe_2–*x*_Mn_*x*_O_4_, *x* = 0.1, 0.2, 0.3; named as NZFM0.1, NZFM0.2, and NZFM0.3; II. Ni_0.5_Zn_0.5–*x*_Mn_*x*_Fe_2_O_4_, *x* = 0.1, 0.2; named as NZM0.1F and NZM0.2F) are prepared through wet ball milling method to address mass production of absorbers and subsequently subjected to a two-step controlled annealing process that generates about 35% porosity in the final system. Moreover, our frequency range of interest, 100 MHz to 9 GHz, covers L-, S-, and C-bands completely, focusing on today’s extensively used frequency regions.^30^ Through this comparative study of tailoring dielectric and magnetic parameters and losses, Ni_0.5_Zn_0.4_Mn_0.1_Fe_2_O_4_ is found to exhibit an optimal excellent reflection loss (RL) of ∼−50.2 dB (i.e., 99.999% EMI shielding) with a broad total effective bandwidth (BW) (RL < −10 dB, i.e., absorption >90%) of 6.8 GHz for a thickness of only ∼6 mm. Moreover, we have explored microwave absorption properties of the studied scalable materials without employing any polymer matrix that is usually utilized to prepare composite EMAs. The polymer/ferrite composites are less durable and have limitations in ferrite content and therefore can possess compromised microwave absorption efficiency.^7^ Simultaneously, the one-step annealed solid NZM0.1F sample is found to possess much lower RL ∼ −28.8 dB than the porous NZM0.1F, which clearly demonstrates the impact of porosity in absorbing EM waves. A distinct enhancement in the attenuation constant (α) is observed from ∼217 Np/m (for NZF) to 301 Np/m (for NZFM0.3) with site-dependent Mn doping. Further, angle-dependent RL study reveals NZM0.1F to be incident EM wave angle insensitive (RL < −10 dB) until 50°, which assures Ni_0.5_Zn_0.4_Mn_0.1_Fe_2_O_4_ as a highly promising low-cost, nontoxic, and stable EMA suitable for practical high-frequency applications. A schematic representation, illustrated in Figure 1, summarizes possible mechanisms behind effective EM wave absorption in porous Mn-substituted NZF absorbers, which makes them extremely suitable for applications in high-frequency devices.

## II Experimental Section

### II.I Synthesis Procedure

Mn-doped Ni–Zn ferrite samples are synthesized by a wet ball milling procedure followed by a two-step heat treatment. Analytical grade (>99% pure, Alfa Aesar) NiO, ZnO, MnO_2_, and Fe_2_O_3_ are taken in stochiometric ratios as precursors, and ethanol is added as a solvent. These powders are hand-mixed for homogeneity and transferred to a steel jar (Retsch) with steel balls as milling media at 1:10 wt %, followed by a continuous milling with speed of 350 rpm with reversals at every 30 min and for 16 h in a planetary ball mill (PM100, Retsch). After that, the liquid mixture is dried at 70 °C for 8 h, and the initial powder is collected after grinding it to avoid any agglomeration. This powder is then calcined for 3 h at 1100 °C in an ambient atmosphere to obtain the stable spinel phase of the ferrites.^31^ During the heat treatment, all of the oxides decompose and recrystallization occurs with the chemical reaction.^32^ Then the as-prepared powder is precisely pressed to a toroidal core of inner (ID) and outer diameters (OD) of 3 mm and 7 mm, respectively, with a 3-Ton hydraulic press (MSE supplies) applying a pressure of 5 MPa. Next, the resulting cores are sintered in the conventional tube furnace at 1150 °C for 1 h followed by normal furnace cooling. Here, the second step temperature is only increased by 50 °C than the first step while reducing the sintering time sufficiently to prevent further particle size growth.^26^ Therefore, this specific heat treatment procedure can retain finite porosity in ferrite cores while maintaining strength and durability. A set of highly dense solid NZM0.1F core samples is prepared following single-step heating of raw ball-milled powder core at 1150 °C for 3 h to compare its EM wave absorption properties with the porous one. This preparation process is schematically illustrated in Figure S1 of the Supporting Information.

### II.II Characterizations

Phase and structural studies of the final samples are inspected by a PANalytical Empyrean X-ray diffractometer (XRD) with Co Kα radiation (λ = 1.7890 Å) and the XRD patterns are converted to Cu Kα (λ = 1.5406 Å) by X’Pert HighScore Plus for further analysis. Thereafter, structural analyses are performed in FullProf_Suite. The relative density and porosity of the samples are calculated based on Archimedes’ principle. Further porosity analysis is performed by X-ray microcomputed tomography (CT) technique using a Bruker SkyScan 1272 scanner. Morphologies and elemental analysis of the samples are investigated employing an FEI Apreo scanning electron microscope (SEM) and the coupled energy-dispersive X-ray (EDX) spectroscopy detector. ImageJ software is used for further pore size and particle size estimation. Magnetic measurements are performed using a vibrating sample magnetometer (VSM) (Lake Shore-8604) in a maximum applied field of 17 kOe at room temperature. Moreover, the microwave properties of the samples are measured with a Keysight E5080A vector network analyzer and a coaxial airline (Maury Microwaves: 2653S3.12) within the frequency range of 0.1–9 GHz. Each 7:3 mm (OD/ID) core sample is inserted in the “port one” end of the coaxial airline, and calibration is performed using the 85032F kit before measurements. Further analysis of the measured two-port scattering (S) parameters is performed with the polynomial fit model of the Nicholson–Ross–Weir algorithm based on the transmission line technique using “N1500A-001 materials measurement suite.”

## III Results and Discussion

### III.I Structure and Morphology

**Figure 2:**
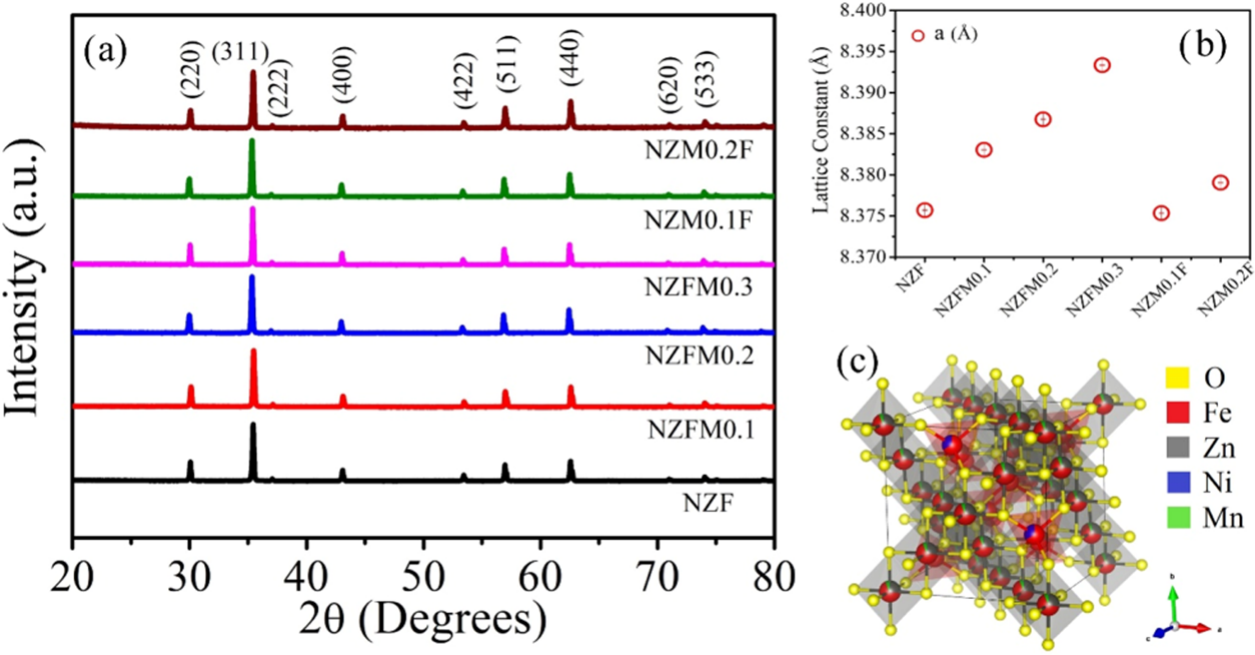
(a) X-ray diffraction plots with identified planes for all of the studied samples at room temperature, (b) variation of lattice constants with samples, and (c) crystal structure representation for NZFM0.3 from VESTA.

XRD patterns of the studied Mn-substituted NZF samples (Ni_0.5_Zn_0.5_Fe_2–*x*_ Mn_*x*_O_4_, *x* = 0.1, 0.2, 0.3; as NZFM0.1, NZFM0.2, and NZFM0.3; and Ni_0.5_Zn_0.5–*x*_Mn_*x*_Fe_2_O_4_, *x* = 0.1, 0.2; as NZM0.1F and NZM0.2F) are displayed in Figure 2(a) and confirm single-phase spinel face-centered cubic structure (space group: *Fd*3̅*m*) as reported in the literature (JCPDS file no. 08–0234 for NZF). Further, Rietveld profile refinements for the XRD plots are performed (shown in Figure S2 of the Supporting Information), where the scale factors, cell, fwhm, peak shape parameters, and atomic occupancies are refined. Estimated lattice constant (*a*) values from refinement for the samples are plotted in Figure 2(b). The obtained “*a*” values show an obvious enhancement with larger size Mn^2+^ (ionic radius: 0.82 Å) doping in place of comparatively smaller size Zn^2+^ (0.74 Å) and Fe^3+^ (0.64 Å)^33^ ions. A representative crystal structure for mixed spinel NZFM0.3 is prepared in VESTA software with the respective refined XRD data and presented in Figure 2(c).

**Figure 3:**
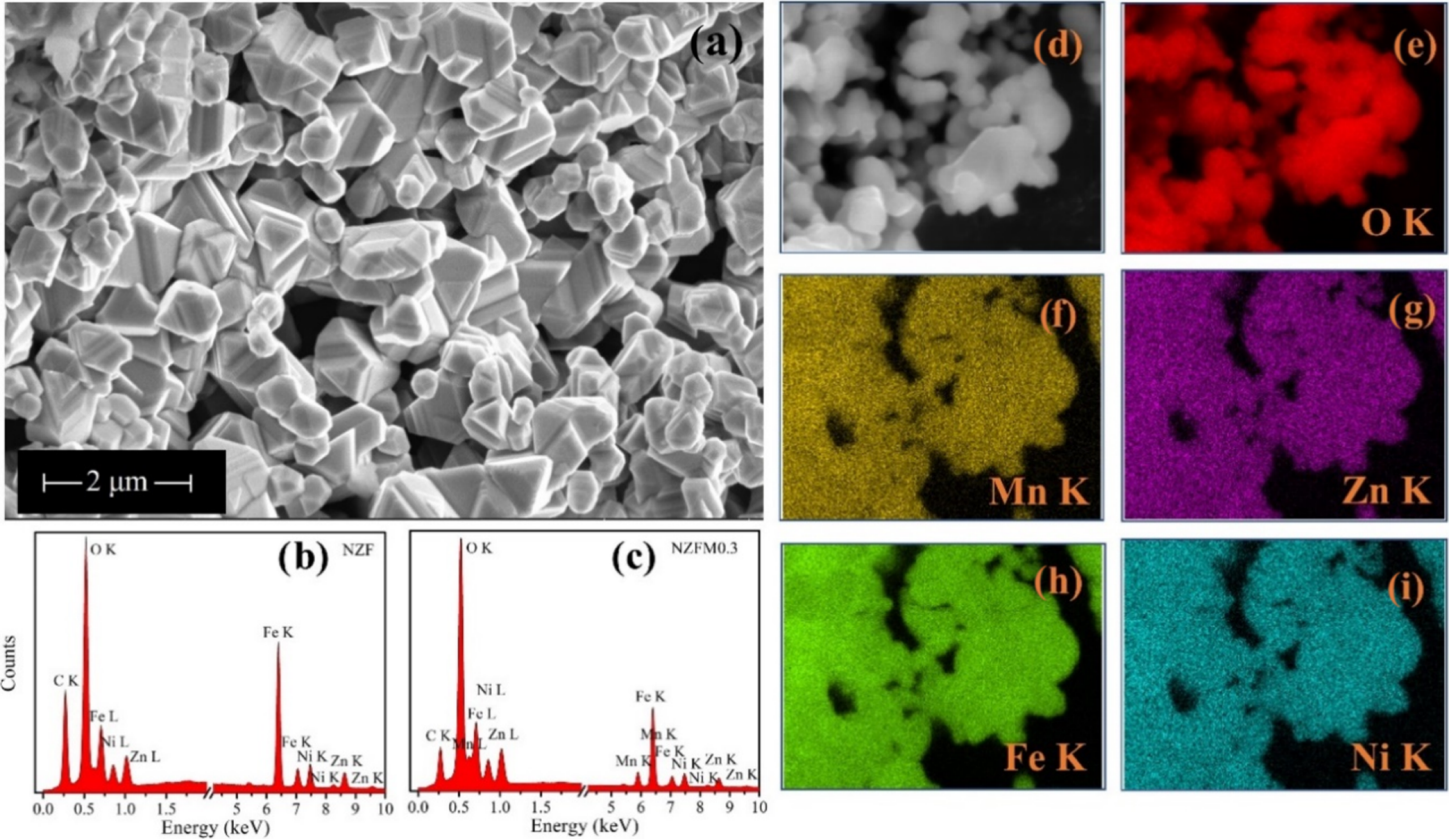
(a) FESEM micrograph of NZFM0.2 core, EDX spectra of samples (b) NZF and (c) NZFM0.3; displays additional Mn peak for NZFM0.3, (d) EDX area mapping for NZFM0.3 sample area; showing homogeneous distribution of constituent elements: (e) O, (f) Mn, (g) Zn, (h) Fe, and (i) Ni.

FESEM micrograph of NZFM0.2, shown in Figure 3(a), illustrates the microstructure and morphology of the samples where the presence of comparatively smaller particles of average size 510 nm with larger size particles of 985 nm is observed. Also, porous spaces of size ∼800 nm can be visualized, which reflects ∼35% porosity in the samples based on estimation from Archimedes’ principle. Both the particle nature and the pore size distribution of the sample sets are found consistent depending on their similar growth mechanism. Figure 3(b,c) shows the EDX spectra of NZF and NZFM0.3, respectively, that depict the absence of Mn contribution in the case of NZF and validate Mn doping. Further, an EDX area mapping for the constituent elements (Ni, Zn, Mn, Fe, and O) is performed in the NZFM0.3 sample, which displays homogeneous elemental distribution throughout the sample.

### III.II Electrical and Magnetic Properties

**Figure 4:**
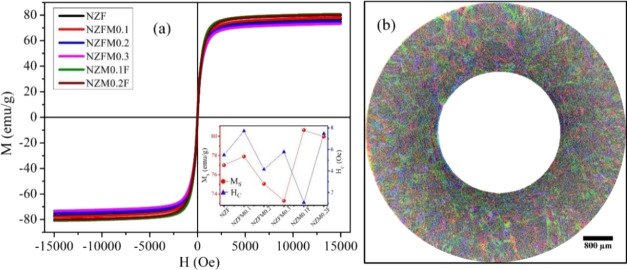
(a) *M*–*H* plots at 300 K for all samples [inset: variation of saturation magnetization (*M*_S_) and coercivity (*H*_C_) with studied samples] and (b) Micro CT scan for cross sections of the NZFM0.3 toroidal sample visualizes colored lines as porous channels in the sample.

Field-dependent magnetization (*M*–*H*) curves at room temperature, displayed in Figure 4(a), exhibit a soft ferrimagnetic nature for the samples. For a clearer view, saturation magnetization (*M*_S_) and coercivity (*H*_C_) values are plotted against respective samples in the inset of Figure 4(a), which shows the variation of *M*_S_ from 73.3 emu/g for NZFM0.3 to 80.7 emu/g for NZM0.1F, and *H*_C_ holds values of less than 7.7 Oe for all of the samples. *M*_S_ is observed to increase with a smaller amount of Mn substitution and then decrease with further Mn content rise. Magnetic contributions from local moments, superexchange interactions, and spin-canting among cations between A-A, A-B, and B–B sites are responsible for the behavior.^33,34^ Additionally, Curie temperature for Mn-doped Ni–Zn ferrite samples is reported as high as ∼600 K suggesting ferrimagnetic properties remain up to that temperature.^26^

Figure 4(b) shows a combination of three transverse cross-sectional tomograms at different layers from a Micro CT scan of the NZFM0.3 toroidal sample. The gray region shows the ferrite material, whereas the colored lines indicate mm-length porous channels in the sample. Here, the visualization of the image and length scale setup is performed with ImageJ analysis. These porous channels can modify the path of the incident microwaves (as for 8 GHz microwave in NZF, λ/4 = 3.1 mm; λ = wavelength) and enhance absorption in this system.^27^

**Figure 5:**
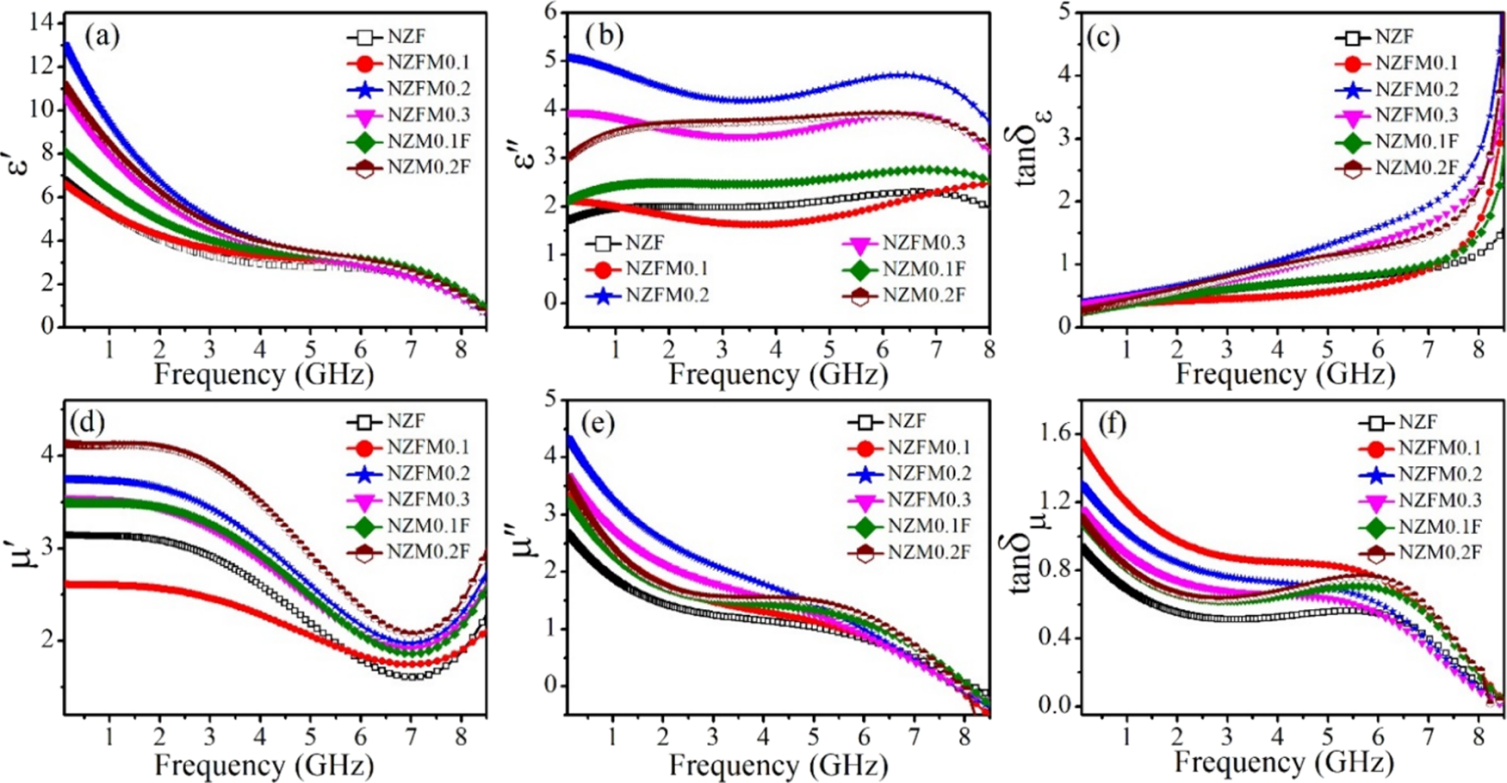
Frequency dispersion of (a) real (ε′) and (b) imaginary (ε″) parts of relative dielectric constants, (c) dielectric loss (tan δ_ε_), (d) real (μ′) and (e) imaginary (μ″) parts of relative permeabilities, and (f) magnetic loss (tan δ_μ_) for the studied samples.

Frequency dependences of real (ε′) and imaginary (ε″) parts of relative dielectric constants for all of the samples are plotted in Figure 5(a,b) in the studied frequency (*f*) region of 0.1–8.5 GHz. For each sample, ε′ decays with increasing frequency, and ε″ shows clear broad humps at decrement slopes of ε′. Here, the dielectric constant originates mainly from the contributions of interfacial and dipolar polarization with additional input from electronic polarization mechanisms. With increasing frequency, dielectric relaxation occurs as these dipoles cannot comply with the electric field and lag the field.^27,35^ Therefore, ε′ falls with rising frequencies along with a strong energy dissipation resulting in ε″ hump, following the Maxwell–Wagner grain–grain boundary model for ferrites.^7^

Moreover, an enhancement for the dielectric constants is observed for the Mn-doped samples reaching a maximum for NZFM0.2, which is associated with the contribution in dipolar polarization from an additional dipole pair Mn^2+^–Mn^3+^ with present other dipoles specially Fe^2+^–Fe^3+^ in between tetrahedral (A) and octahedral (B) sites for NZF system.^36^ Redistribution of cations with larger size Mn doping in NZF also causes strain in the lattice,^37^ promoting A-B intersite Fe^2+^–Fe^3+^ charge hopping, which increases έ value as well.^19^ Further, lattice distortion can augment electron scattering and aid in increasing the interfacial polarization in the system.^38^ The distorted and asymmetric nature of the Cole–Cole plots, i.e., ε″ versus ε′ curves for the samples, shown in Figure S3 (in the Supporting Information) again suggests interfacial polarization between the grains and more than one semicircular arc signifies different dipoles participating in this dielectric relaxation process resulting in non-Debye-type relaxation.^12,39^ Consecutively, dielectric loss tangents, defined as tan δ_ε_ = ε″/ ε′, for all of the samples^40^ are displayed in Figure 5(c) as a function of frequency. tan δ_ε_ is found to show an increasing trend with the frequency for the studied samples. At the same time, with increasing EM wave frequency, grain boundary impedance gradually falls and the grain-to-grain charge hopping starts to take place, which boosts conductivity and corresponding conduction loss in the samples.^7,41^ Frequency dependences for the ac conductivity of the samples are shown in Figure S4. Therefore, total electrical loss in such a system is associated with both polarization and conduction losses.^3^

Frequency variation of real (μ′) and imaginary parts (μ″) of relative permeability of the samples are plotted in Figure 5(d,e) as a function of frequency from 0.1 to 8.5 GHz. μ′ values are observed to decrease up to a certain *f*, whereas μ″ displays characteristic broad resonance peaks for the samples at around 5.5 GHz corresponding to the downward slope frequency of μ′. Permeability dispersion of NZF is associated with domain-wall motion, spin rotation, and natural resonance according to the modified Landau–Lifshitz–Gilbert (LLG) equation.^23,25,27^ Moreover, initial permeability for polycrystalline samples follows the Globus equation, μ′ ∝ (*M*_S_^2^*d*/*K*^1/2^), where *K* is the magneto-crystalline anisotropy constant, and *d* is the grain size.^42^ Here, the μ values increase with Mn substitution and reach a maximum for NZM0.2F with moderate magnetization and magnetic anisotropy.

Next, frequency-dependent magnetic loss tangents (tan δ_μ_ = μ″/ μ′) for all of the samples^40^ are displayed in Figure 5(f), where tan δ_μ_ is observed to decline with *f* for all of the samples with a distinct broad resonance around 5.5 GHz. It is well known that dynamic magnetic loss mostly arises from domain-wall resonance, magnetic hysteresis, eddy current effects, and natural and exchange resonance.^6,19^ The first two contributions are less effective in this study due to the presence of a low magnetic field and high frequency.^12^ For NZF samples, strong EM absorption through magnetic loss around 5.5 GHz arises as a consequence of the natural magnetic resonance (NMR) and is also related to the exchange interaction between neighboring grains.^27,43^ NMR is governed by the equation, , where γ is the gyromagnetic ratio (∼2.8 GHz/kOe for ferrites),  is the anisotropic field for the samples, and *f*_r_ is the resonance frequency calculated as 3.8 GHz. Here, *K* is derived from the “law of approach to magnetic saturation” equation.^44^ Hence, contributions in magnetic loss can be attributed to magnetic resonance at comparatively lower *f*, whereas eddy current loss has a significant role in absorption at much higher frequencies.^12^

### III.III Microwave Absorption Properties

Electromagnetic wave (EM) absorption properties study is strongly correlated with magnetic and electrical properties of a material. Among these properties, reflection loss (RL) gives insights into the material’s efficiency in shielding the reflected waves.^1,45^ This can happen through either wave absorption, i.e., transforming EM wave to heat energy via losses, or transmission of EM wave.^6^ Contextually, proper impedance matching ensures better propagation of EM wave through the material enhancing the chances for losses as well.^35^

**Figure 6:**
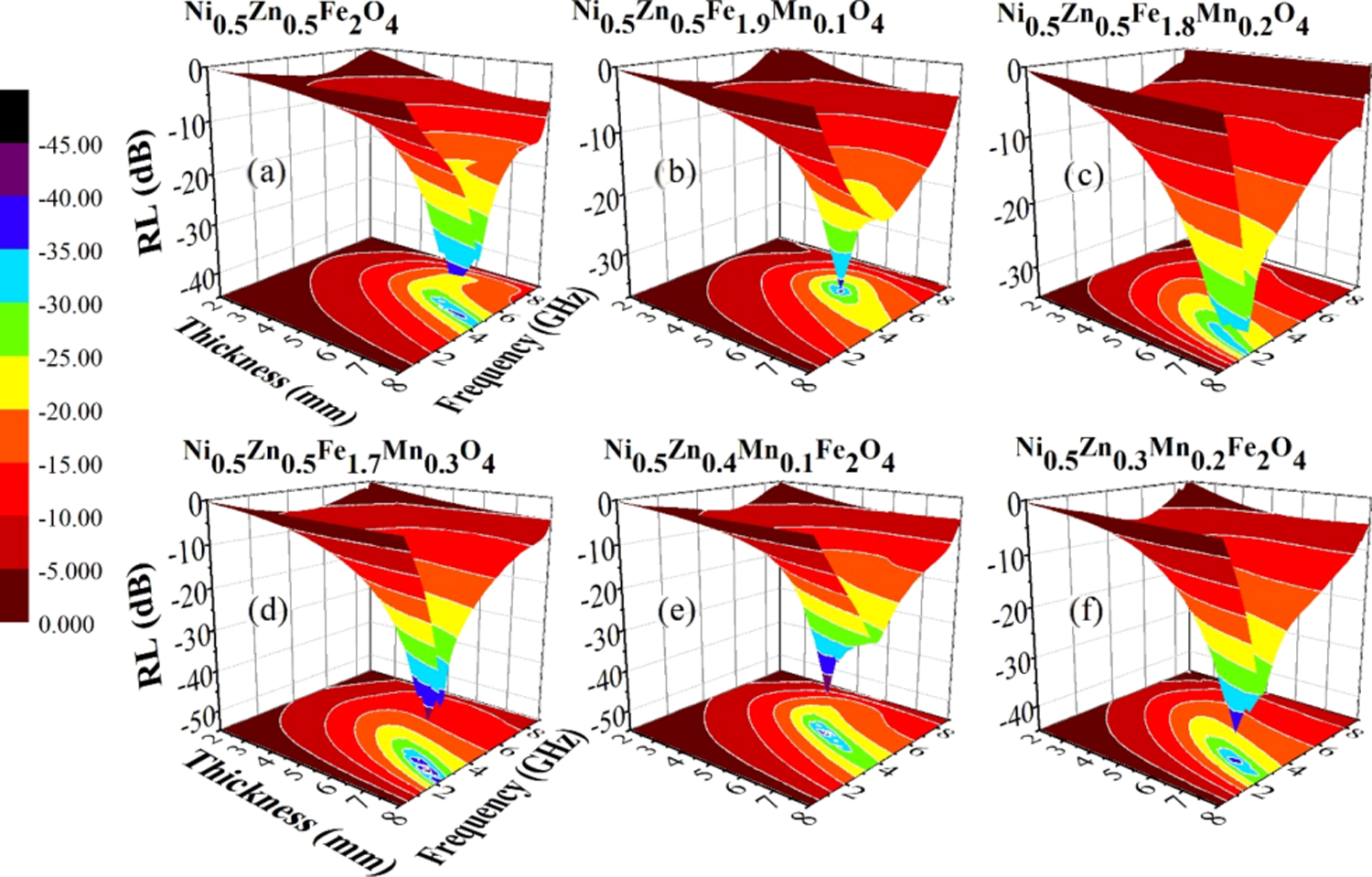
(a–f). 3D representation of RL varying with frequency and thickness for the studied samples.

Frequency-dependent reflection loss (RL) curves for NZF and Mn-doped NZF cores are plotted in Figure 6(a–f) varying their thicknesses from 2 to 8 mm. These figures portray a successful tuning of microwave absorption properties with controlled Mn doping in the Ni–Zn ferrite sample. Here, RL is calculated following these equations^11,46,47^^11,46,47^
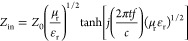
1
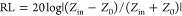
2where μ_r_ (= μ′ – *i*μ″) and ε_r_ (= ε′ – *i*ε″) are the relative permeability and permittivity of the material, *c* is the velocity of light, *t* is the absorber thickness, and *Z*_0_ and *Z*_in_ are the impedance of free space and input impedance of absorber, respectively.

Excellent microwave absorption property in these samples originates from total magnetic and dielectric losses in the material. To enhance the total loss, porosity in the core samples plays a major role by elongating the total path length of the EM wave in the system and introducing additional interfaces so that it gets absorbed to a greater extent.^10,14^ Some recent studies displayed that the generated heat in the system can activate more charge carriers contributing again to the total loss.^4^ Moreover, a thickness-dependent study on EM wave absorption is also carried out for a proper thickness (*t*) design of the NZF absorbers required for practical applications.^3^ Though for a similar *t*, RL peak frequency (*f*_m_) varies with material properties (μ, ε) following the relation, , as interpreted from the quarter wavelength (λ/4) model.^11^

**Figure 7:**
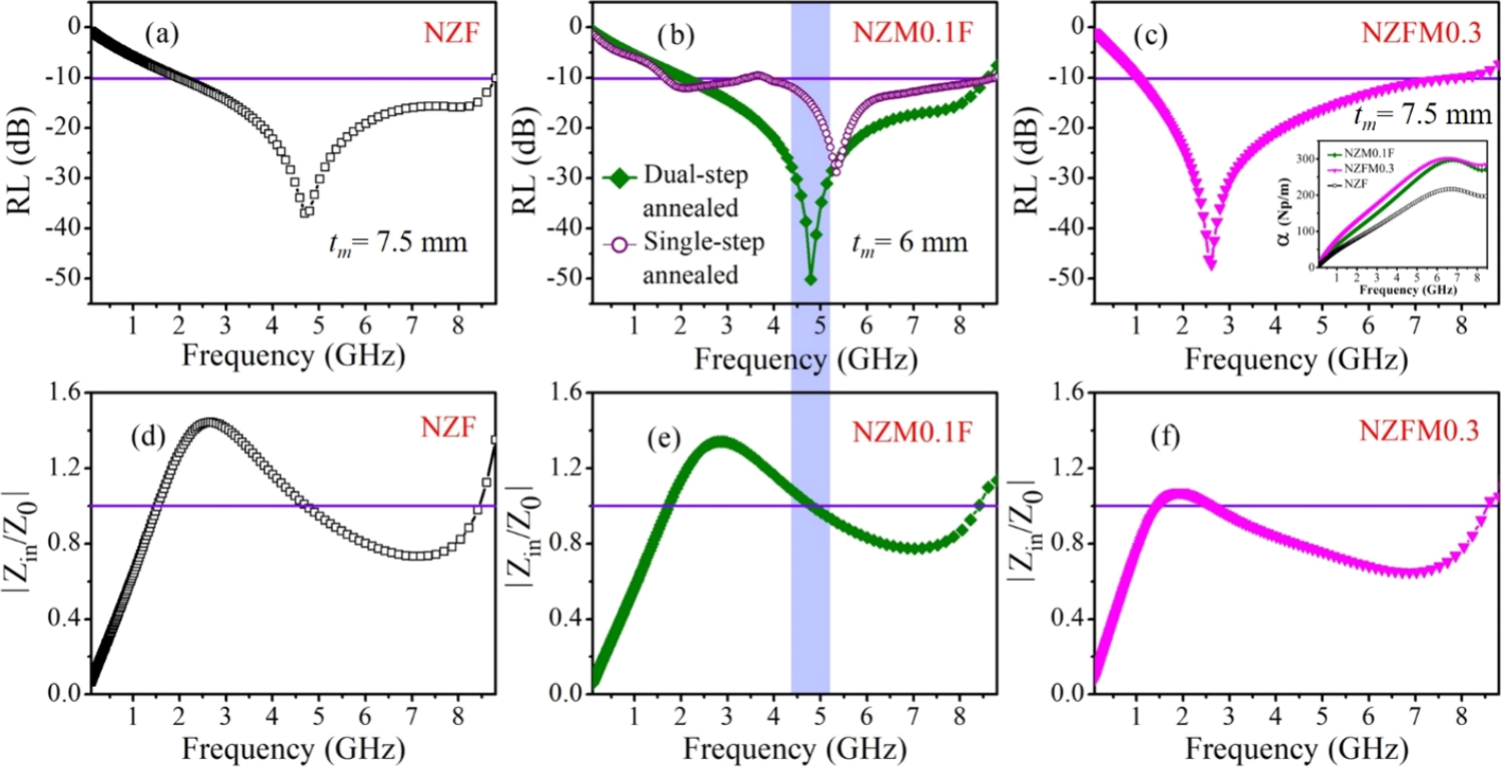
RL vs *f* plots at respective *t*_m_ values for (a) NZF, (b) NZM0.1F and single-step annealed solid NZM0.1F, and (c) NZFM0.3 samples for comparison. Corresponding |*Z*_in_/*Z*_0_| ratio vs frequency plots for (d) NZF, (e) NZM0.1F, and (f) NZFM0.3 [inset of (c)] Frequency-dependent attenuation constant (α) curves for these three samples.

Figure 7(a–c) compares optimum RLs and effective bandwidths (BW) for NZF, NZM0.1F, and NZFM0.3 samples at their respective optimum thicknesses (*t*_m_; the thickness at which RL_max_ is obtained) with corresponding |*Z*_in_/*Z*_0_| versus frequency plots in Figure 7(d–f). RL_max_ values for NZM0.1F and NZFM0.3 are −50.2 dB (*t*_m_ = 6 mm, *f*_m_ = 4.79 GHz) and −47.1 dB (*t*_m_ = 7.5 mm, *f*_m_ = 2.6 GHz), respectively, which is significantly improved from the base NZF one (RL_max_ = −36.9 dB, *t*_m_ = 7.5 mm, *f*_m_ = 4.68 GHz). BW (RL < −10 dB) is also widened in the case of NZM0.1F (6.8 GHz) than NZF (6.5 GHz), which demonstrates NZM0.1F as a broadband EMA. |*Z*_in_/*Z*_0_| vs frequency plots show the best impedance matching is achieved for NZM0.1F sample referred to its broader frequency region in the vicinity of |*Z*_in_/*Z*_0_| = 1.0. This outcome proves that with proper Mn doping in NZF, sufficient dielectric and magnetic losses in addition to favorable impedance matching in NZM0.1F are responsible for its excellent EM wave absorption. Additionally, to observe the effect of porosity in EM wave attenuation, RL is compared for dual-step annealed porous NZM0.1F core (porosity: 35%) with single-step annealed solid NZM0.1F core (porosity: 14%, RL_max_ = −28.8 dB, *t*_m_ = 8 mm, *f*_m_ = 5.35 GHz) in Figure 7(b). This plot signifies the impact of porosity in the system for enhancing wave absorption. Frequency-dependent dielectric constants and permeability values for the solid NZM0.1F core are shown in Figure S5 of the Supporting Information.

Further, the attenuation constant (α) is another important parameter for evaluating EM wave losses in the sample and is expected to be as high as possible. α is associated with microwave loss during transmission through the samples as ∝ e^–*αt*^, are estimated and shown in the inset of Figure 7(c). α is defined in terms of relative permittivity and permeability as^12,46^
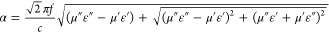
3Here, α is observed to increase with frequency and with increasing values of ε_r_ and μ_r_, α is found to rise from ∼217 Np/m for NZF to 301 Np/m for NZFM0.3 and 296 Np/m for NZM0.1F through controlled Mn substitution.

**Figure 8:**
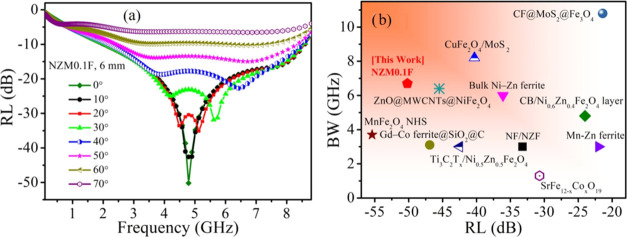
(a) Frequency dispersion curves of RL at different EM wave incident angles. (b) A comparative graph for optimal reflection loss (RL_max_) and bandwidth (BW) of NZM0.1F with other ferrite-based promising microwave absorbers: CuFe_2_O_4_/MoS_2_,^11^ CF@MoS_2_@Fe_3_O_4_,^41^ Bulk Ni–Zn ferrite,^7^ ZnO@MWCNTs@NiFe_2_O_4_,^46^ CB/Ni_0.6_Zn_0.4_Fe_2_O_4_ layer,^40^ MnFe_2_O_4_ NHS,^27^ Gd–Co ferrite@SiO_2_@C,^6^ NF/NZF,^47^ Mn–Zn ferrite,^20^ Ti_3_C_2_T_*x*_/ Ni_0.5_Zn_0.5_Fe_2_O_4_,^35^ and SrFe_12–*x*_Co_*x*_O_19_.^16^

Next, an EM wave incident angle (θ)-oriented microwave absorption study for the optimized NZM0.1F (*t* = 6 mm) sample is simulated based on transmission line theory considering θ dependencies for *Z*_in_ and RL,^16,48^ as shown in Figure 8(a). With the increase of θ from 0 to 70°, the wave absorption efficiency tends to decrease throughout the frequency region, resulting from the gradually mismatched impedance at oblique incidences, accompanied by additional peaks. Additionally, reduced EM wave amplitudes with higher angles cause insufficient excitation to the sample developing less losses in it.^22^ However, higher values of ε and μ dilute the effect of oblique incidence.^16^ Interestingly, here the studied NZM0.1F sample demonstrates incident angle insensitiveness up to 50° with RL < −10 dB (>90% shielding), which establishes it as an all-around EM wave absorbing material.

The maximum RL and BW obtained in this study are comparable to other promising ferrite-based microwave absorbers as presented in Figure 8(b). Though, these chemically and physically optimized, scalable NZF samples serve as a simple single-component MW absorber and extend the limits for its commercial implementations.

## IV Conclusions

In summary, a magnetic–dielectric balance is successfully achieved from 0.1 to 9 GHz region by regulating Mn substitution in Ni–Zn ferrite, which suggests chemical tuning to be an effective strategy to enhance EM wave absorption. Sufficient porosity is incorporated in the samples with a controlled two-step heating procedure preceded by a scalable ball milling process, which results in a lightweight EMA and contributes to increasing the total losses through multiple EM wave scatterings in the samples. With synergistic dielectric, conduction, and magnetic losses in the system and most favorable impedance matching with |*Z*_in_/*Z*_0_| nearly equals to 1, NZM0.1F, among the six studied sample sets (NZF, NZFM0.1, NZFM0.2, NZFM0.3, NZM0.1F, and NZM0.2F) reaches to the strongest microwave absorption with RL = −50.2 dB (shielding >99.999%) at 4.79 GHz with a broad effective BW of 6.8 GHz at only 6 mm thickness. Cationic redistribution and microstructural strain play important roles in varying the electrical polarization and magnetic interactions in the system. This composition-dependent study reveals a significant improvement in RL and attenuation constant (α) from −37 to −50.2 dB and from 217 to 301 NP/m, respectively. Furthermore, the optimum NZM0.1F sample shows an incident wave angle insensitivity of up to 50°, which describes the all-around performance of the absorber. Therefore, an excellent RL and broad BW observed in the NZM0.1F sample illustrate it as a stable, feasible, and cost-effective promising EMA material for various high-frequency applications.

## Data Availability

The data that support the findings of this study are available from the corresponding authors upon reasonable request.

## Supporting Information Available

Sample preparation schematic diagram; Rietveld profile refinement of XRD plots; Cole–Cole plots for the samples; frequency dependence of ac conductivity for the samples; real and imaginary parts of permittivity and permeability vs frequency of single-step annealed solid NZM0.1F core sample (PDF)
