# Supplementary material for: All-Around Electromagnetic Wave Absorber Based on Ni–Zn Ferrite
Source: ACS Appl Mater Interfaces. 2024 Jun 20;16(26):33846–54. doi: 10.1021/acsami.4c06498 (PMC11231975; doi:10.1021/acsami.4c06498)
Supplement: Supplementary file 1 — am4c06498_si_001.pdf [file am4c06498_si_001.pdf]

Supporting Information

All-around electromagnetic wave absorber based on Ni-Zn ferrite

Dipika Mandal*, Bishal Bhandari, Suraj V. Mullurkara, and Paul R. Ohodnicki*

Department of Mechanical Engineering and Materials Science, University of Pittsburgh, Pittsburgh, PA 15260, USA

*E-mails: dim65@pitt.edu (DM) and pro8@pitt.edu (PRO)

1. Flow diagram for the synthesis and preparation procedure for the Ni-Zn ferrite based electromagnetic wave absorbers.

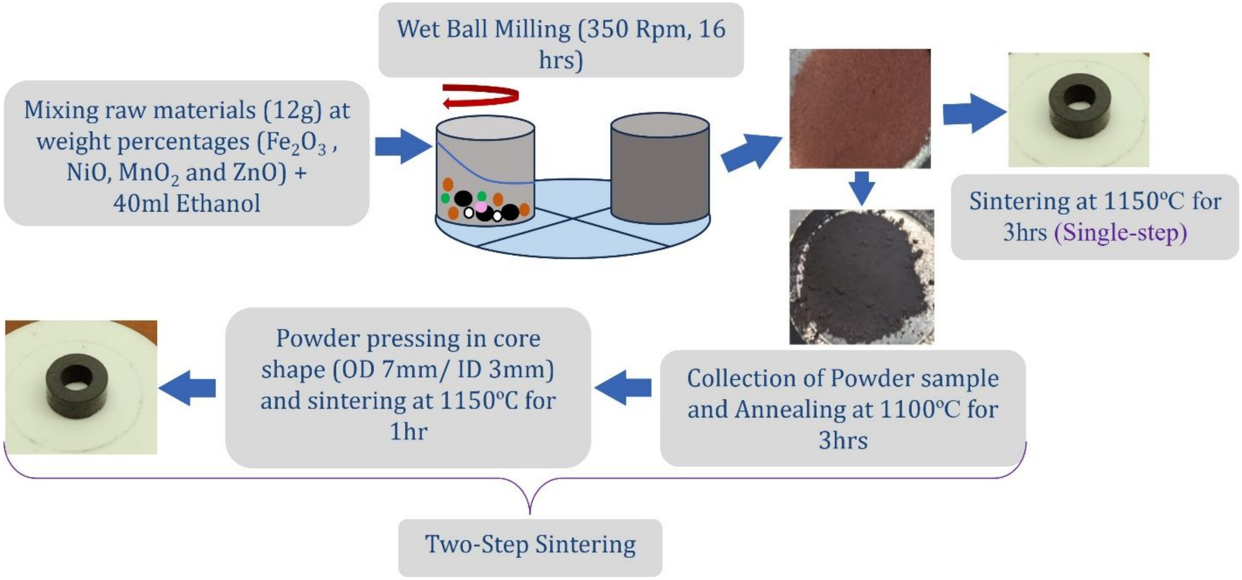

Figure S1: Schematic diagram of preparation process of Ni-Zn ferrite-based EMA

2. The XRD plots of the studied samples with Rietveld profile refinement.

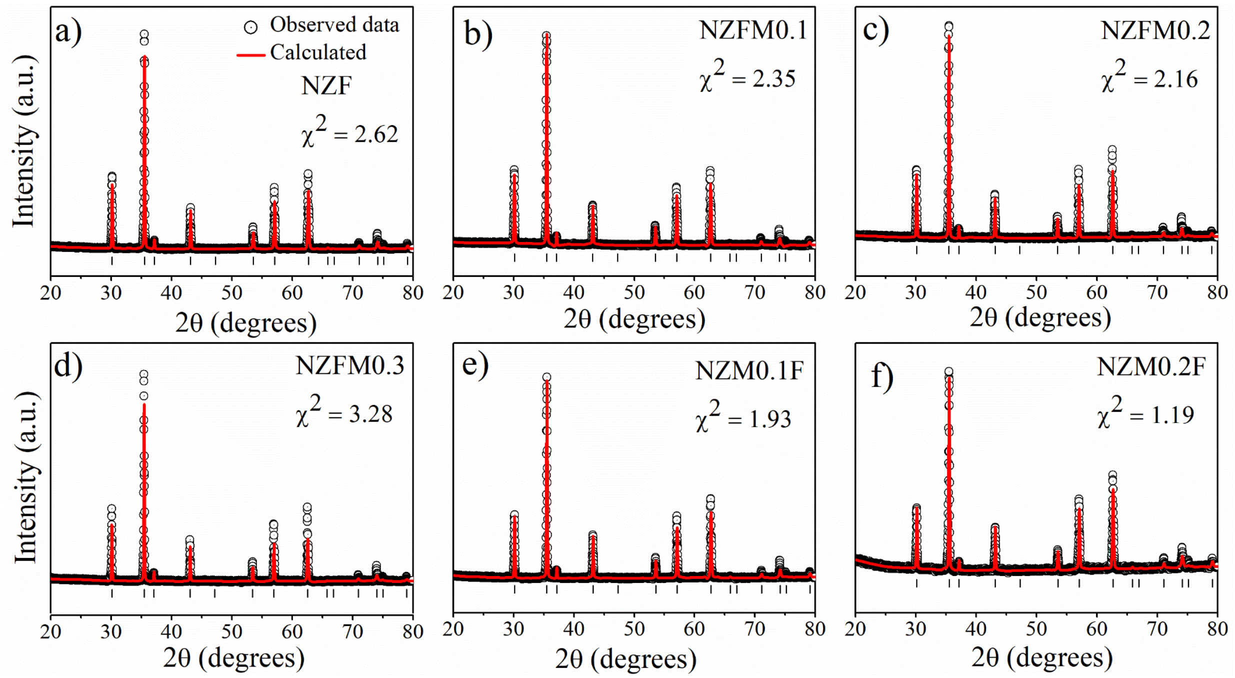

Figure S2(a-f): XRD data for the samples and their Rietveld profile refinements

The peak shape is described as 'Thompson-Cox-Hastings pseudo-Voigt*Axial divergence asymmetry' formulation whereas scale factors, cell, FWHM and shape parameters, atomic positions with respective occupancies are refined. These patterns confirm single-phase spinel face centered cubic structure within resolution of XRD measurement as reported in the literature. The structure refinement ensured good values for the reliability factors with a goodness of fit χ^2 varying from 1.19 to 3.28. The refined lattice parameters (a) are plotted in **Fig. 2(b)** of the main text.

3. Cole-Cole plots.

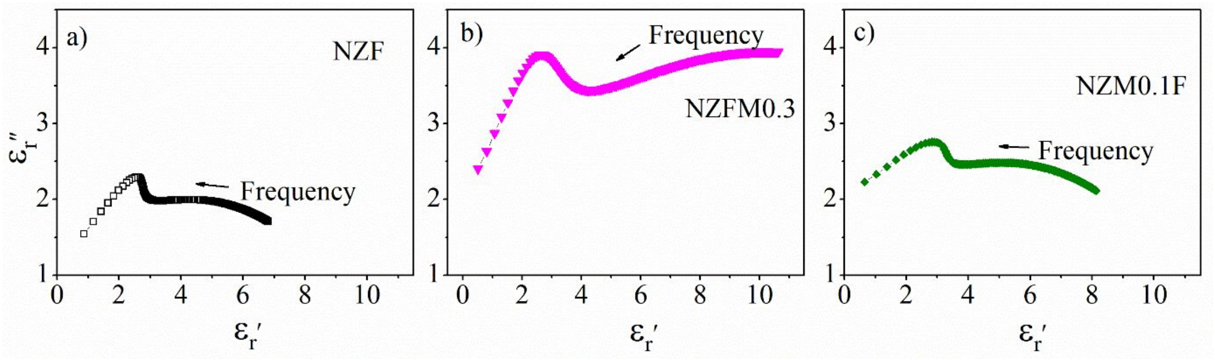

Figure S3: Cole-Cole plots for samples a) NZF, b) NZFM0.3, c) NZM0.1F

Cole-Cole plots for the samples, NZF, NZFM0.3, and NZM0.1F are illustrated in **Fig. S3**. According to Debye theory, relation between ε_r'' and ε_r', can be deduced as follows,

$$\left(\varepsilon_r' - \frac{\varepsilon_s+\varepsilon_\infty}{2}\right)^2 + (\varepsilon_r'')^2 = \left(\frac{\varepsilon_s+\varepsilon_\infty}{2}\right)^2 \quad \text{(S1)}$$

where ε_s and ε_∞ stands for static permittivity and high-frequency finite permittivity, respectively [1]. Curves following this equation form semicircular arcs representing each dielectric relaxation.

These plots also follow Maxwell-Wagner conductive grain–resistive grain boundary model for interfacial polarization in ferrites, where with increase in frequency, permittivity values decrease. Here, the ε_r'' versus ε_r' plots in the studied frequency region for the samples are consisted of more than one broad distorted asymmetric semi-circular arcs, which suggest the relaxation processes are of modified Debye type. Role of more than one kind of dipolar relaxations in dielectric relaxation and presence of different charge carriers in cases of both NZF and Mn-substituted Ni-Zn ferrite are responsible for this nature [2].

4. AC conductivity vs. frequency for the studied samples.

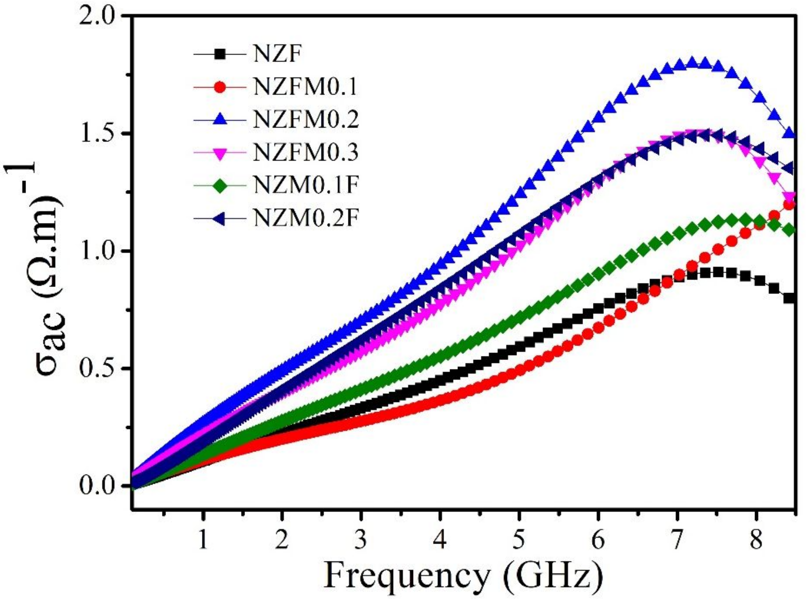

Figure S4: Frequency dependence of ac conductivity for all the studied samples shows increment in conductivity with increasing frequency

5. Dielectric constants and magnetic permeability for the solid NZM0.1F sample

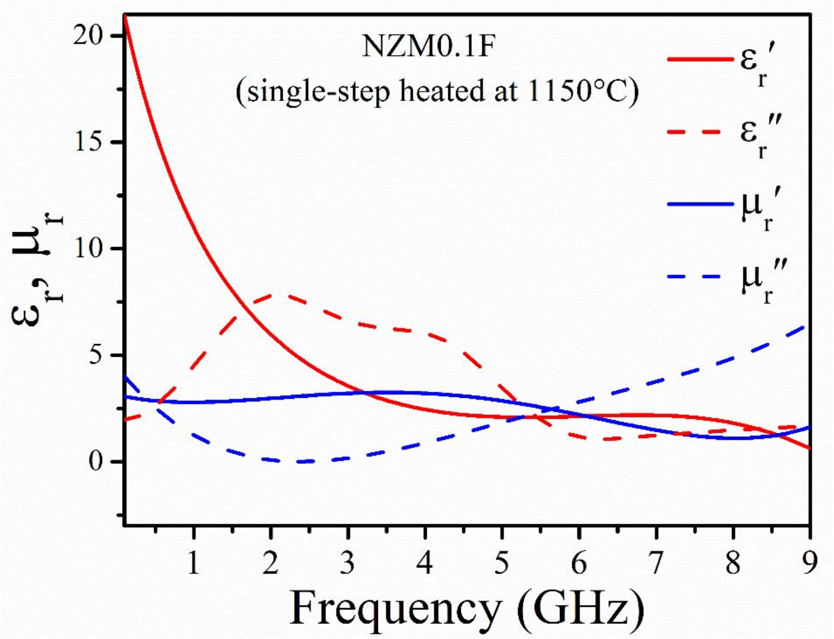

Figure S5: Real and imaginary parts of permittivity and permeability vs. frequency of solid NZM0.1F core sample single-step annealed at 1150ºC.

Dielectric constants and magnetic permeability for the solid NZM0.1F sample are plotted at the studied frequency region which follows the similar natures as the porous Ni-Zn ferrite samples in this work. However, as for higher density core, polarizability and magnetization of the sample increases, **Fig. S5** shows comparatively higher values of permittivity and permeability for this sample.

References:

(1) Gao, J.; Ma, Z.; Liu, F.; Weng, X.; Meng, K. Preparation and Microwave Absorption Properties of Gd–Co Ferrite@silica@carbon Multilayer Core–Shell Structure Composites. *Chem. Eng. J.* **2022**, *446* (P4), 137157. https://doi.org/10.1016/j.cej.2022.137157.

(2) Sun, X.; Li, Y.; Huang, Y.; Cheng, Y.; Wang, S.; Yin, W. Achieving Super Broadband Electromagnetic Absorption by Optimizing Impedance Match of RGO Sponge Metamaterials. *Adv. Funct. Mater.* **2022**, *32* (5). https://doi.org/10.1002/adfm.202107508.

(3) Derakhshani, M.; Taheri-Nassaj, E.; Jazirehpour, M.; Masoudpanah, S. M. Structural, Magnetic, and Gigahertz-Range Electromagnetic Wave Absorption Properties of Bulk Ni–Zn Ferrite. *Sci. Rep.* **2021**, *11* (1), 1–13. https://doi.org/10.1038/s41598-021-88930-0.
